# Indian Consensus on the Role of Amitriptyline in Migraine Prophylaxis

**DOI:** 10.7759/cureus.54270

**Published:** 2024-02-15

**Authors:** Sumit Singh, A V Srinivasan, Tapas K Banerjee, Kanharam N Patel, Snehal S Muchhala, Bhavesh P Kotak

**Affiliations:** 1 Neurology, Artemis Agrim Institute of Neurosciences, Gurugram, IND; 2 Neurology, Dr. MGR Medical University, Chennai, IND; 3 Neurology, National Neurosciences Centre, Kolkata, IND; 4 Neurology, Dr. Reddy’s Laboratories, Hyderabad, IND; 5 Medical Affairs, Dr. Reddy’s Laboratories, Hyderabad, IND

**Keywords:** quality of life, amitriptyline, consensus, headache, migraine disorders

## Abstract

Migraine is a globally prevalent neurological disorder. Amitriptyline, a tricyclic antidepressant, has shown potential as a prophylactic treatment for migraine; however, its role as a first-line medication has been debated. A modified Delphi method was used to develop consensus statements on migraine and its management. The literature review identified knowledge gaps, and two survey rounds were conducted among a panel of experts. Consensus was reached for 12 out of 23 initial survey questions, whereas no consensus was reached for four questions after the deliberation in the second round. The results showed that migraine is highly prevalent among women aged 15-35 years in India. Amitriptyline is an effective monotherapy for prophylactic migraine management, with a recommended initial dose of 5-10 mg. A gradual titration over six months achieves optimal results. Amitriptyline is also safe for managing catamenial migraine and can be used at lower doses during pregnancy to alleviate symptoms. The outcomes of this study emphasize that amitriptyline should be considered as a primary prophylactic treatment for migraine because of its efficacy and safety. The evidence-based consensus achieved is intended to serve as guidance for healthcare practitioners in India, and it is anticipated that such adoption will lead to improvement in patient outcomes and an enhancement in the quality of life for those affected by migraines.

## Introduction

Migraine is a neurological disorder distinguished by severe, pulsating unilateral headache accompanied by symptoms such as nausea, photophobia, phonophobia, and vomiting [1]. Between 1990 and 2019, the worldwide age-standardized prevalence of migraine increased by 1.7%. In 2019, the prevalence was 1.1 billion cases, which was associated with 525.5 years lived with disability (YLDs) per 100,000 population [2]. Migraine can substantially impair patients’ quality of life and finances [3]. Based on a review of epidemiological studies conducted in 2007, it was observed that in India, migraine led to losses in paid work (2%), household work (2%), and gross domestic product (1.7%) [4]. Migraine ranked second in contributing to global neurological disability-adjusted life years (DALYs) in 2016, accounting for 16.3% of DALYs [2]. Comorbidities associated with migraine include psychiatric disorders such as anxiety and depression [5].

Treatments for migraine include preventive medications, acute migraine therapy, and a range of nonpharmacological therapies [6]. Episodic and chronic migraine often require preventive treatment such as tricyclic antidepressants (TCAs); beta-blockers; anticonvulsants; calcium channel blockers; antidepressants that act as serotonin-norepinephrine reuptake inhibitors, selective serotonin reuptake inhibitors, and noradrenergic and specific serotonergic antidepressants; angiotensin II receptor blockers; botulinum toxin; and calcitonin gene-related peptide (CGRP) monoclonal antibodies [7,8]. TCAs were among the first migraine prophylactics to be discovered. Amitriptyline was discovered in the late 1950s and approved by the United States Food and Drug Administration (FDA) in 1961 [7]. Amitriptyline use is primarily indicated for depression, fibromyalgia, and chronic neuropathic pain. Other indications include anxiety, insomnia, post-traumatic stress disorder, irritable bowel syndrome, interstitial cystitis, and postherpetic neuralgia [9].

Amitriptyline plays a role in migraine management by inhibiting the neurotransmitter 5-hydroxytryptamine (5-HT) or serotonin in the synaptic cleft. Its status as the first drug of choice in prophylactic therapy for migraine is debated in India. While several countries use amitriptyline as the first choice of medication, the European Headache Federation, the European Academy of Neurology, and the American Headache Society (AHS) recommend it as a second-line medication [7].

While some studies have demonstrated the efficacy of amitriptyline in migraine prophylaxis, others have highlighted challenges and limitations, reflecting a lack of consensus regarding its use [7]. In India, the prophylactic management of migraine with amitriptyline lacks expert recommendations.

Objective

The objectives of this study were to develop guidelines for migraine prophylaxis including the indications for the use of amitriptyline, dosage of amitriptyline in migraine prevention, and duration of therapy.

## Materials and methods

A modified Delphi method was employed for the development of the expert recommendations. Extensive literature searches were conducted on electronic databases, such as PubMed and Embase, to identify relevant articles published in English between 2007 and 2023. These searches helped identify gaps in knowledge and unmet needs related to migraine management across different parts of the world. The findings from the searches were submitted to a team of three steering committee members (SCMs)/core panel members who consented to participate in the survey. The SCMs formulated a list of 23 pre-consensus statements based on the results of these searches. These statements were further circulated in the first round of the survey among an advisory panel that comprised 22 experts from different parts of India with >25 years of experience in clinical practice and research on migraine management. The responses from the experts were collected anonymously. An ethical clearance from the internal review board was not deemed necessary as it was considered to be minimal risk. The initial discussion with the panel members included an overview of the background, purpose, and scope of the paper. The roles and responsibilities of the panel members were described. An overview of the literature search used for the pre-consensus statements was elucidated. A proposed timeline for the development of the consensus was chalked out during the meeting.

Two survey rounds were conducted to gather consensus from the expert panel.

**Figure 1:**
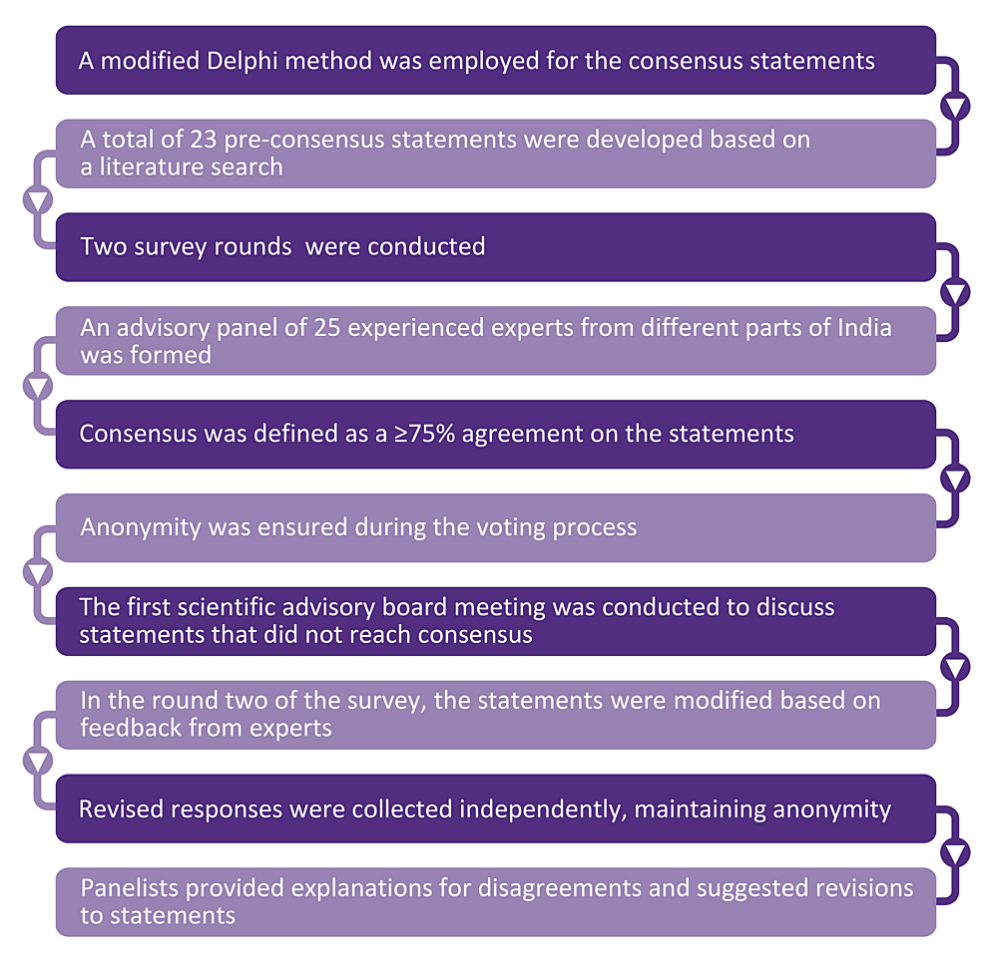
Summary of the methodology.

The questionnaire used in this study had two parts: one included statements on the burden and pathophysiology of migraine, and the other contained statements on the prophylactic/preventative treatment of migraine using amitriptyline. For the first round of the survey, a total of 23 statements were mailed to the experts using an online survey platform, the responses for which were collected based on a Likert scale. The results of the e-consensus were computed. A high consensus was defined a priori as ≥75% agreement on a given answer. A moderate consensus was considered when the agreement was between 55-74% among the experts. Statements that received an agreement below 55% were considered as not having reached a consensus. Round 1 of the e-consensus survey was followed by the first scientific advisory board meeting (round 2) to discuss the statements. For the second round, the statements that did not reach a consensus in round 1 were modified. Additionally, some of the statements that had reached a high consensus in round 1 were reworded for clarity. A re-poll was conducted, and the revised responses were collected. After detailed deliberations by the experts, the collated responses were used to derive recommendations on the prophylaxis of migraine in India, with a focus on amitriptyline. A summary of the methodology is depicted in Figure 1.

## Results

In the initial round of the modified Delphi method, a consensus was achieved for 12 out of 23 questions. The 11 questions that did not reach consensus were revised and included in the second round of the survey. However, even after the second round of deliberations, a consensus could not be reached for four questions. The final consensus statements are presented in Table 1, providing an overview of the agreement among the expert panelists during the consensus-building process.

**Table 1:** Consensus levels in the first and second rounds of the survey. ADR: adverse drug reaction; NR: not reframed; OD: once daily; TTH: tension-type headache

Sl. no.	First round of statements	Consensus reached after the first round	Reframed statements	Consensus reached after the second round
1.	Migraine is the most common type of headache seen in clinical practice.	Consensus	All headaches are migraines unless proven otherwise; migraine often overlaps with TTH.	Consensus
2.	Migraine is more commonly seen in the age group of 15–35 years.	Consensus	Migraine is more commonly seen in the productive age group of 15–35 years as compared to older individuals.	Consensus
3.	Migraine is more pronounced in women.	Consensus	NR	
4.	The episodic type of migraine is more frequently observed in patients.	No consensus	At the first encounter, it was found that episodic migraine is more common in patients.	No consensus
5.	Recognition of comorbid conditions in migraine is important as they can influence the treatment choice.	Consensus	It is essential to recognize comorbid conditions as they influence the choice of treatment.	Consensus
6.	More than 10% of the patients with episodic migraine transform into chronic migraine.	No consensus	More than 10% of episodic migraine cases transform into chronic migraines.	No consensus
7.	Frequent and severe attacks of migraine can be a debilitating factor for patients’ quality of life.	Consensus	NR	
8.	Prophylactic therapy may decrease the frequency, severity, and duration of migraine attacks; increase responsiveness to acute migraine therapy; and improve quality of life.	Consensus	NR	
9.	Amitriptyline has been one of the mainstays of migraine prophylaxis for over 35 years and is currently one of the most used drugs as first-line monotherapy.	No consensus	Amitriptyline may be used as first-line monotherapy, and any variation may be based on individual clinical practice.	Consensus
10.	The usual starting dose of amitriptyline prescribed in clinical practice for the preventive treatment of migraine is 5–10 mg.	Consensus	The starting dose of amitriptyline prescribed in clinical practice for preventive treatment of migraine is 5–10 mg. Amitriptyline is started at low doses for migraine prophylaxis to avoid excessive sedation.	Consensus
11.	The dosing frequency and duration for amitriptyline in preventive therapy for migraine is OD at night or bedtime.	Consensus	Amitriptyline is prescribed OD at night or bedtime for preventive therapy of migraine to give a better sedative/hypnotic effect and to prevent drowsiness the next morning.	Consensus
12.	Dose escalation of amitriptyline may be considered.	Consensus	Practitioners may increase amitriptyline doses.	Consensus
13.	Dose escalation of amitriptyline is done after two weeks of migraine prophylaxis in case of low response to the initial dose.	No consensus	Dose escalation of amitriptyline is done post-two weeks of migraine prophylaxis when there is a low response to the initial dose.	Consensus
14.	The maximum tolerated dose of amitriptyline in migraine is 75 mg OD.	No consensus	The maximum tolerated dose of amitriptyline in the prophylactic treatment of migraine is 75 mg OD.	No consensus
15.	Patients can see an appreciable clinical improvement in their migraine (reduction in frequency, severity, and duration) in two to four weeks.	No consensus	Patients on amitriptyline show appreciable clinical improvement in migraine (reduction in frequency, severity, and duration) in two to six weeks.	Consensus
16.	The duration of therapy recommended for amitriptyline in the prophylactic treatment of migraine patients for optimal results is more than 4 months.	No consensus	The duration of therapy recommended for amitriptyline in the prophylactic treatment of migraine patients for optimal results is six months.	Consensus
17.	The ADRs seen in migraine patients on amitriptyline prophylactic therapy usually wane off with time and do not require discontinuation of therapy.	Consensus	The ADRs seen in migraine patients on amitriptyline prophylactic therapy usually wane off with time and do not require discontinuation of therapy in all patients.	Consensus
18.	In clinical experience, ADRs are managed for patients on amitriptyline prophylaxis through the continuation of amitriptyline therapy with patient counseling or reduction of dosage.	No consensus	ADRs are managed for patients on amitriptyline prophylaxis through patient counseling and continuation of amitriptyline therapy or subsequent dose reduction.	No consensus
19.	Amitriptyline can be prescribed for migraine prophylaxis in patients without depression.	Consensus	NR	
20.	Amitriptyline is preferred in menstrual migraine.	No consensus	Amitriptyline can be prescribed for menstrual migraine.	Consensus
21.	Amitriptyline can be prescribed for migraine in pregnant women.	No consensus	There are class C recommendations for amitriptyline. Thus, amitriptyline can be given in low doses for migraine during pregnancy if needed.	Consensus
22.	On a scale of 1 to 10, amitriptyline as a prophylactic therapy in migraine patients in terms of effectiveness, adverse events, and adherence can be rated more than 6.	No consensus	Amitriptyline as a prophylactic therapy in migraine can be scored as 6-10 for patients in terms of effectiveness, adverse events, and adherence.	Consensus
23.	Amitriptyline scores highest when it comes to its use in migraine with comorbid depression, insomnia, neuropathic pain, and fibromyalgia.	Consensus	NR	

## Discussion

Epidemiology

Headache is a poorly understood condition with inadequate data on the disease burden. In India, very few studies have reported the disease burden of headaches; therefore, a comprehensive assessment of this condition is warranted. Globally, headache disorders affect around 50% of adults, of which >30% can be attributed to migraine. Headaches lasting >15 days per month affect 1.7%-4% of the adult population worldwide. Recent epidemiological research conducted in the capital region of Delhi reported a one-year migraine prevalence of 27.2%, underscoring the substantial migraine burden in this area [3]. The panel said that 20%-30% of patients visiting their clinical practice experienced a chief complaint of headache. The expert panel agreed that unless proven otherwise, all headaches are migraines. However, many patients with migraine also suffer from coexisting medication-overuse headaches. Furthermore, the study revealed coexistence/overlap between migraine and tension-type headaches (TTH).

The expert panel opined that the prevalence of migraine is higher in the age group of 15-35 years. Stovner et al. indicated the greatest proportion of migraine-associated YLDs in both genders in the age group of 15-49 years [10]. Unanimously, experts agreed that migraine is more prevalent among women. Stovner et al. reported a global standardized migraine prevalence of 14.4%, observed more often in women (18.9%) than in men (9.8%) [10]. Experts could not concur on the predominant type of migraine observed by doctors during their first encounters with patients complaining of headaches. It was noted that patients often initially present with episodic migraine; however, this would progress to a chronic state by the time the patient consulted the specialists. Therefore, the management of both types is dependent on the expertise and experience of the specialists. A common opinion was that untreated episodic migraine may become chronic. The panelists noted that India’s transition rate from episodic to chronic migraine has surpassed 2.5%-3.0%, as per a study by Bigal et al. [11]. Furthermore, the panelists emphasized that maintaining a migraine diary for at least six months can help doctors and patients develop individualized prophylactic treatment regimens in the Indian scenario. The Chronic Migraine Epidemiology and Outcomes study conducted in 2012 found that 83.6% and 13.7% of patients consulted physicians for episodic and chronic migraine, respectively [12].

Diagnosis

With the absence of biochemical markers for migraine detection, diagnosis relies primarily on physical examination, patients' clinical history, and the exclusion of alternative headache disorders. The clinical diagnostic criteria include symptoms such as nausea, vomiting, photophobia, phonophobia or osmophobia, headaches lasting 4-72 hours, episodic headaches, and unilateral headaches [6,13]. The expert panel also identified these as symptoms that distinguished migraine from other types of headaches. The expert panel unanimously reached a consensus about the importance of recognizing comorbidities associated with migraine, notably depression and anxiety, as these significantly impacted treatment selection. Important migraine triggers include stress, skipping meals, inadequate sleep, hormonal changes, and weather changes [14]. Recognizing comorbidities in individuals with migraine helps optimize drug choices and improve treatment outcomes [6,13].

The expert panel did not reach a consensus on the percentage of patients developing chronic migraine, despite optimal treatment. However, the prevailing evidence indicates that about 3% of cases with episodic migraine progress into chronic migraine within a year, highlighting the crucial role of healthcare professionals in monitoring and managing patients along with the impact of individual clinical experiences in recognizing this progression [15]. The expert panel unanimously agreed on the substantial impact of frequent and severe migraine attacks on patients’ quality of life, leading to substantial debilitation. Migraine is a prevalent and incapacitating condition that detrimentally impacts multiple domains of functioning, such as interpersonal relationships, job performance, educational accomplishment, and overall quality of life not only during acute migraine attacks but also persistently between episodes [16].

Treatment

Migraine management necessitates a proper diagnosis before commencing treatment. Early intervention with efficacious medications is crucial in managing acute migraine attacks [1]. The panel suggested prophylactic therapy for patients who experience frequent migraine attacks that last >24 hours and cause major daily disruptions. The expert panel also mentioned that the primary determinants for selecting prophylactic therapy for migraine are therapy efficacy, side effects, and the presence of comorbidities. Amitriptyline benefits migraine patients with TTH, depression, neuropathic pain, and insomnia. The panel predominantly agreed on the efficacy of amitriptyline in the management of insomnia-related migraines. The panelists agreed that amitriptyline has been extensively studied in migraine therapy and cited as the first-line option for preventive treatment in >20 studies (2018-2020). While AHS and European guidelines designate it as grade B or second-line therapy. The European guidelines also mention amitriptyline as a reasonable choice for first-line therapy in patients with migraine having comorbid depression [7].

This study reached a consensus regarding the potential benefits of prophylactic therapy in reducing the frequency, severity, and duration of migraine attacks while also improving responsiveness to acute treatment and overall quality of life. Ha et al. [17] reported the efficacy of preventive medication in reducing the frequency, severity, and related distress of episodic migraine, while also improving the quality of life and preventing its progression to chronic migraine. Indications for preventive therapy include frequent headaches, debilitating symptoms, and medication overuse. Identifying and managing triggers such as environmental, dietary, and behavioral factors and the use of nonpharmacological approaches are valuable strategies in migraine prevention [18]. The panel agreed that nonsteroidal anti-inflammatory drugs (NSAIDs) combined with an antiemetic constitute the first-line treatment for acute migraine. While amitriptyline is favored as a potential prophylactic first-line monotherapy for migraines, the panel acknowledged possible variations in drug selection because of clinical practices. Although amitriptyline shows promise as a prophylactic treatment for migraines, its efficacy requires further research. Amitriptyline, as monotherapy or in combination with other treatments, is an important drug for the prevention and management of migraines [9]. The preferred drug of choice currently used in combination with amitriptyline, as agreed upon by most of the panel members, is propranolol. The panel members concurred that using amitriptyline in combination with propranolol can have a synergistic effect in treating migraine.

The expert consensus agreed that the recommended initial dose of amitriptyline for the preventive treatment of migraines is 5-10 mg, emphasizing low starting doses to minimize the risk of excessive sedation. The expert panel unanimously recommended dose escalation strategies, particularly favoring bedtime dosing, to enhance the sedative and hypnotic effects while minimizing morning drowsiness. To optimize the safety and effectiveness of migraine treatment, a conservative approach involving the initiation of therapy with low drug doses is recommended for all prescribed medications, regardless of the complexity of the condition [9]. Empirical evidence supports initiating amitriptyline at lower doses, specifically 10 mg per night, and adopting a gradual dose escalation approach [19]. 

The consensus supported amitriptyline dose increment in migraine prophylaxis after two weeks of inadequate response to the initial dose. Experience from clinical practice supports starting with the lowest effective dose and gradually increasing it every two to four weeks until the desired therapeutic effects are achieved or until adverse effects develop [17].

Consensus was not reached on the maximum tolerated dose of amitriptyline being 75 mg once daily (OD) for the prophylactic treatment of migraine. A widely recommended maximum dose of amitriptyline for pain treatment is 75 mg per day [20]. Dosage determination considers comorbidities; those with sole migraines may benefit maximally from 25 mg, while the presence of depression or neuropathic pain warrants higher doses of 75-100 mg. The expert panel identified amitriptyline, propranolol, flunarizine, and topiramate as the preferred choices of drugs in the prophylaxis of migraines while recognizing amitriptyline's unique attributes. CGRP inhibitors, despite being a significant advancement in migraine treatment, have certain limitations that affect their usage. The availability and high cost of these drugs in India are a primary concern, making them less accessible compared to traditional prophylactic agents. Their use is often limited to cases where other preventive medications have either failed or are contraindicated [6,21]. Owing to these considerations, other preventive drugs are often used and preferred in many cases, especially for patients with less severe migraine symptoms or where cost and convenience are significant factors.

Patients on amitriptyline typically observe clinical improvement within two to six weeks, marked by reduced migraine frequency, severity, and duration. A six-month duration is advised for optimal prophylactic results. Diener et al. reported optimal results with amitriptyline after four months of administration and recommended that amitriptyline be used for an adequate duration of time for managing chronic migraines [22]. Early treatment discontinuation, based on perceived inefficacy, may affect the efficacy of preventive therapy [6].

Experts agreed that adverse drug reactions (ADRs) from amitriptyline prophylactic therapy in migraine patients tend to diminish over time, often not requiring treatment discontinuation. ADRs vary with treatment duration and patient condition [9]. For ADR management, the panel suggested continued therapy and patient counseling as ADRs gradually diminish over time. Additionally, reducing amitriptyline dosage might be preferred over drug discontinuation. However, management practices vary based on individual factors, treatment response, and stage. Brueckle et al. reported a link between drug interactions and coexisting conditions/concomitant medications [23]. Anticholinergic ADRs of amitriptyline include constipation, dryness of mouth, tachycardia, agitation, confusion, delirium, increased risks of falls, hallucinations, and cognitive issues. Amitriptyline-related ADRs include vision problems, dizziness, sedation, and sexual dysfunction; however, not all of them stem from muscarinic inhibition [23]. Drowsiness and anticholinergic effects such as constipation and urinary retention are common among migraine patients on amitriptyline prophylaxis. Longitudinal studies are necessary to comprehensively explore amitriptyline's potential adverse effects in migraine treatment [9].

The expert panel agreed on amitriptyline's potential for prophylactic migraine treatment in nondepressed patients. Amitriptyline, a TCA, shows promise because of its efficacy in migraine management, extending its utility beyond patients with depression. The Canadian guidelines for primary care management of headaches in adults recommend amitriptyline as the first-line prophylactic medication for migraine [24]. Guidelines by the German Migraine and Headache Society and the German Society of Neurology support amitriptyline use in migraine prevention [22]. The US Headache Consortium also categorizes amitriptyline in group 1 medications with proven high efficacy and mild-to-moderate adverse effects [25]. However, it is classified as a level B drug for migraine prophylaxis by the AHS and the American Academy of Neurology, indicating its "probably effective" status even though it has not received specific FDA approval for this indication. Additionally, in Europe, amitriptyline is recognized as a second-choice medication for the treatment of migraines [7].

The expert panel concurred on amitriptyline use in the management of menstrual migraines. Devika et al. [5] highlighted that TCAs, specifically amitriptyline and nortriptyline, are commonly prescribed for migraine management. These medications are utilized as part of a comprehensive approach that involves both acute and preventive treatment strategies [5]. The panelists suggested that drugs such as acetazolamide, topiramate, NSAIDs, clobazam, and longer-acting triptans are effective in managing catamenial migraine. If amitriptyline has no impact on menstrual cycles and is safe, it can be prescribed continuously for 6 months for catamenial migraine, especially if other treatments are ineffective.

According to the expert panels’ consensus, amitriptyline can be prescribed at low doses for the treatment of migraines during pregnancy as it is classified as relatively safe (class C). However, nonpharmacological approaches are preferred over preventive therapy during pregnancy [26]. The expert panel recommended initiating amitriptyline at a low dose for migraine prophylaxis to allow for careful monitoring of its effects and to avoid excessive sedation, while still achieving effective migraine prevention.

A consensus was established on amitriptyline’s efficacy in migraine management; it was rated between 6 and 10 for effectiveness, adverse events, and patient adherence. Amitriptyline can reduce the number of monthly migraine days by >50%. However, it is crucial to acknowledge the limited treatment duration assessed in the available studies, which necessitates further investigation into the long-term effectiveness of amitriptyline.

The expert panel agreed that amitriptyline demonstrated the highest efficacy in treating migraine with comorbidities such as depression, insomnia, neuropathic pain, and fibromyalgia. Amitriptyline is optimal for preventing migraines in patients with depression, as its dosage (75-150 mg/day) aligns with depression management [22]. For migraine patients with comorbid depression, unresponsive to low amitriptyline doses, dose escalation was recommended by most panelists as a suitable approach.

Numerous studies highlight the importance of early identification and personalized treatments for managing migraines. Research suggests amitriptyline to be a promising drug for migraine prevention and improving patients’ quality of life.

The limitation of this study was that since the study followed a consensus approach, the results were subjective and based on expert opinions. The consensus process can be influenced by the individual experiences and biases of the experts involved, which must be considered when assessing the generalizability of the findings or their ability to fully represent the spectrum of clinical practice. Further research is needed to validate these results. Moreover, the panelists highlighted the need for additional real-world data regarding the epidemiology of chronic and episodic migraines in India, which points to a gap in the current understanding specific to the Indian context. Furthermore, there are varying perspectives among the panelists, indicating that there was not complete agreement on all aspects. This suggests a need for more comprehensive discussions and possibly a more in-depth analysis of the issues at hand.

Recommendations

The panelists expressed the importance of generating additional real-world data on the epidemiology of chronic and episodic migraines in India. Given the varying perspectives among the panelists, further comprehensive discussions are required to address the utilization of amitriptyline for managing migraines during pregnancy. This study highlights existing knowledge gaps in migraine management, particularly regarding the use of amitriptyline in different scenarios. The results derived from the consensus contribute to the body of literature by offering evidence-based recommendations for migraine management, particularly in the context of Indian settings. Developing guidelines for its prophylactic use and sharing them through educational workshops among neuro-physicians is crucial. Additionally, exploring novel formulations of amitriptyline, such as sustained-release tablets, could enhance patient convenience and compliance by reducing the number of tablets needed to be administered.

## Conclusions

This study critically evaluated the role of amitriptyline as a prophylactic treatment for migraine. Despite the ongoing debates about its positioning as a first-line therapy, this consensus-based research underscores the effectiveness and safety of amitriptyline in treating migraines. Starting with an initial dose of 5-10 mg and careful titration over six months has been identified as an optimal approach for prophylactic management. Notably, amitriptyline has also been recognized for its safety in managing catamenial migraines and its applicability at lower doses during pregnancy to mitigate symptoms. This evidence-based consensus aims to provide practical guidance for healthcare practitioners across India, advocating for the adoption of amitriptyline as a primary prophylactic treatment for migraines.
